# Lipid metabolism marker CD36 is associated with ^18^FDG-PET/CT false negative lymph nodes in head and neck squamous cell carcinoma

**DOI:** 10.3389/fonc.2023.1156527

**Published:** 2023-05-03

**Authors:** Xiaoyan Meng, Jingjing Sun, Feng Xu, Zhonglong Liu, Yue He

**Affiliations:** ^1^ Department of Oral Maxillofacial & Head and Neck Oncology, Shanghai Ninth People’s Hospital, Shanghai Jiao Tong University School of Medicine, Shanghai, China; ^2^ College of Stomatology, National Center for Stomatology, National Clinical Research Center for Oral Diseases, Shanghai Key Laboratory of Stomatology, Shanghai, China; ^3^ Department of Oral Pathology, Shanghai Ninth People’s Hospital, Shanghai Jiao Tong University School of Medicine, Shanghai, China; ^4^ Department of Nuclear Medicine, Shanghai Ninth People’s Hospital, Shanghai Jiao Tong University School of Medicine, Shanghai, China

**Keywords:** ^18^FDG-PET/CT, head and neck cancer, lymph node metastasis, CD36, metabolism

## Abstract

**Background:**

Lymph node metastasis frequently occurs in head and neck squamous cell carcinoma (HNSCC) patients, and [^18^F] fluorodeoxyglucose positron emission tomography with computed tomography (^18^FDG-PET/CT) examination for lymph node metastasis could result in false negativity and delay following treatment. However, the mechanism and resolution for ^18^FDG-PET/CT false negatives remain unclear. Our study was aim to found biomarkers for false negativity and true positivity from a metabolic perspective.

**Methods:**

Ninety-two patients diagnosed with HNSCC who underwent preoperative ^18^FDG-PET/CT and subsequent surgery in our institution were reviewed. Immunohistochemistry (IHC) examinations of glucose metabolism (GLUT1 and GLUT5), amino acid metabolism4 (GLS and SLC1A5), and lipid metabolism (CPT1A and CD36) markers were conducted on their primary lesion and lymph node sections.

**Results:**

We identified specific metabolic patterns of the false-negative group. Significantly, CD36 IHC score of primary lesions was higher in false-negative group than true-positive group. Moreover, we validated pro-invasive biological effects of CD36 by bioinformatics analysis as well as experiments. Conclusion: IHC examination of CD36 expression, which is a lipid metabolism marker, in primary lesions could distinguish HNSCC patients’ lymph nodes false negatives in ^18^FDG-PET/CT.

## Introduction

Head and neck squamous cell carcinoma (HNSCC) is the sixth most common cancer worldwide (1). A large proportion of HNSCC patients suffer from lymph node metastasis whose first-line therapy is dissection surgery (2). Therefore, preoperative evaluation of lymph nodes is important. However, some clinical N0 patients showed lymph node metastasis according to the final pathological report, which referred to false negativity. The false negativity of lymph nodes may delay the patient’s treatment plan.

With the development of examination methods, positron emission tomography (PET) with computed tomography (CT) using [^18^F] fluorodeoxyglucose (FDG) is widely performed for the detection of lymph node metastases in HNSCC. However, ^18^FDG-PET/CT still exhibits a false negative rate of 10.5%-37.0% (3–7). The mechanisms for false negativity remain unclear, and the resolution to improve false negativity has not yet been developed.

Since ^18^FDG-PET/CT was performed using ^18^FDG, an analog of glucose that is abnormally taken up by cancer cells (8), we hypothesized that the glucose metabolic status as well as other metabolic activities of the examined region could be causative factors for false negativity. Therefore, we chose six metabolism-related molecules (GLUT1, GLUT5, GLS, SLC1A5, CPT1A, and CD36) to represent the three main metabolic activities and examined their expression levels by immunohistochemistry (IHC). GLUT1 is the main glucose transporter on tumor cells (9). GLUT5 is the isoform of GLUT1 and mediates fructose uptake (10). GLS is the key enzyme for glutamine metabolism (11), and SLC1A5 controls glutamine uptake in various tumor types (12). CD36 controls the uptake of fatty acids (13), and CPT1A mainly mediates fat oxidation in tumor cells (14).

By comparing these metabolic markers expression between primary lesions and metastatic nodes, we concluded specific metabolic activity patterns of the false negative group. More significantly, we found that CD36 and GLS in primary lesions could be promising biomarkers for distinguishing false negative nodes. This would be of great importance for improving outcomes of HNSCC patients.

## Materials and methods

### Patient cohort

We conducted a retrospective cohort study to investigate the expression of 6 metabolic markers (GLUT1, GLUT5, GLS, SLC1A5, CPT1A, and CD36), ^18^FDG-PET/CT diagnosis and pathological diagnosis in a sample of HNSCC patients consecutively admitted to our hospital. Our analysis included patients between 1 January 2018 and 31 December 2021. They underwent ^18^FDG-PET/CT examination and then surgery in our hospital, the interval of which was less than 3 months.

**Figure 1:**
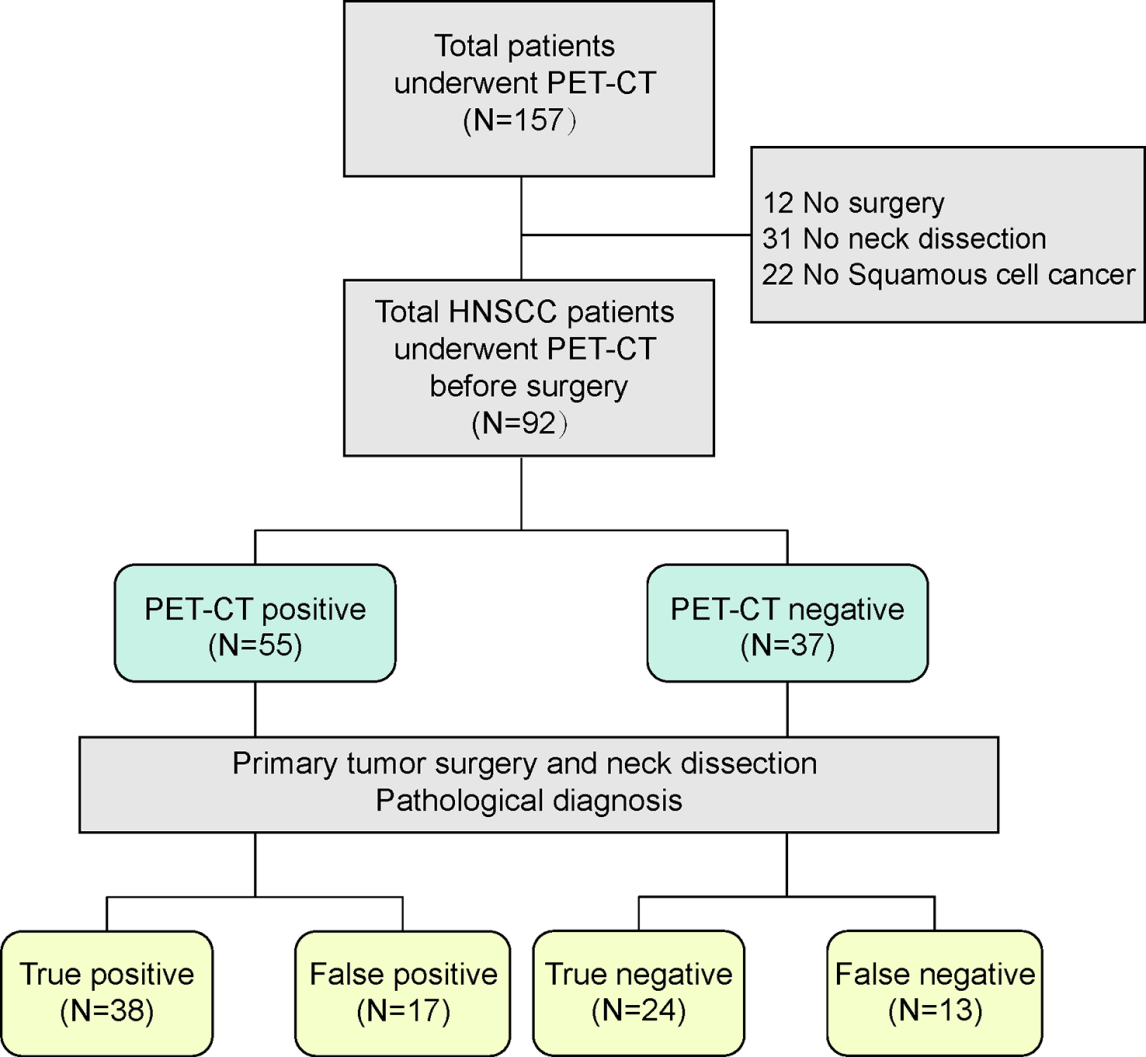
Flowchart of the study population, inclusion and exclusion criteria.

The inclusion criteria were 18-90 years of age, primary lesion in head and neck region, squamous cell carcinoma for final pathological diagnosis, and ^18^FDG-PET/CT examination before surgery. The exclusion criteria were as follows: no squamous cell carcinoma for final pathological diagnosis, preoperative adjuvant treatment, a history of radiation in the head and neck area, a history of chemotherapy or immunotherapy before surgery, and a delay of more than 6 weeks between ^18^FDG-PET/CT and surgery. The final study population for statistical analysis was 92 patients (**Figure 1**).

### PET/CT image acquisition and lymph node evaluation

This study examined all ^18^FDG-PET/CT acquisition with the SIEMENS biograph mCT Flow PET/CT system (Simens Medical Solutions United States, Inc.). After at least 6 h of fasting, the patient was intravenously administered a standard dose (3.7 MBq/kg) of ^18^F-FDG, followed by image acquisition 60 min later, from the thigh to the head. Whole-body noncontrast enhancement CT scanning protocols were as follows: 120 kVp, 30–170 mAs adjusted to the patient’s body weight and with a section width of 3 mm and collimation of 0.75 mm. Data were reconstructed into coronal, sagittal, and transverse sections and a 3-dimensional rotating projection. Visible lesions with increased tracer uptake were identified, and their ^18^F-FDG uptake was quantified. The maximum standardized uptake value (SUV_max_) was semiquantitatively analyzed according to the equation SUV 5A/(ID/LBW), where A is the decay-corrected activity in tissue (in MBq/mL), ID is the injected dose of ^18^F-FDG (in MBq), and LBW is the patient’s lean body weight.

For lymph node metastasis, all images were reviewed by experienced nuclear medicine physicians and abnormal ^18^F-FDG uptake was determined. The definition of abnormal ^18^F-FDG uptake was an accumulation outside the normal anatomic structures, or as higher uptake than background activity or asymmetric uptake, which are not normally seen. Image interpretation was based on visual and semiquantitative analyses of abnormal increased focal ^18^F-FDG uptake, but strict standardized uptake value cutoffs were not used.

### Radiographic and pathological interpretation for lymph nodes metastasis

We reviewed radiographic as well as pathological results of patients’ lymph nodes. Since it is difficult to assure resected nodes exactly correspond to those identified in the image, we focused on overall neck side rather than single lymph node. Neck sides could be classified into four groups; true negative (TN), false positive (FP), false negative (FN), and true positive group (TP). TN side means that there is neither radiographic abnormal lymph nodes nor pathological metastasis on this side; FP means that there exists radiographic abnormal nodes but pathological evaluation shows lymph nodes on this side are all negative; FN and TP are opposite situations to FP and TN, respectively. Moreover, since our objective is to predict ^18^FDG-PET/CT and pathological results based on primary tumor metabolic features and to avoid FN results disturbing clinical decisions, therefore, we make statistics on single patient (we cannot match one primary tumor with two neck sides in the meantime) and set a relative strict standard. To be more specific, for those undergo unilateral neck resection, we classified them based on their resection side; for those have bilateral neck resection, we identified them as “FN” or “FP” patient as long as they have one “FN” or “FP” neck side (There is no patient with one “FN” side and one “FP” side in our cohort). In this way, we are able to integrate one patient’s radiological and pathological information for further analysis with IHC results.

### IHC examinations and histopathological evaluations

IHC staining was performed according to the staining protocol. In brief, tissue specimens were fixed in 4% formaldehyde and embedded in paraffin. Then, the tissue sections were deparaffinized, rehydrated, and incubated overnight at 4°C with primary antibodies against GLUT1 (ab115730, 1:250, Abcam), GLUT5 (ab76316, 1:250, Abcam), GLS (ab156876, 1:100, Abcam), SLC1A5 (ab237704, 1:1000, Abcam), CPT1A (ab234111, 1:1000, Abcam), and CD36 (ab252922, 1:250, Abcam). After washing, the bound antibody was detected with horseradish peroxidase (HRP)-conjugated secondary antibody at 37°C for 30 min and then visualized using a DAB kit. IHC staining is shown in **Figure S1**.

The pathological evaluation was conducted by pathologists. For H&E staining of primary tumor and lymph node slides, they were examined with items like depth of invasion (DOI), pathological stage, differentiation status, neuron/vessel invasion status, and extranodal extension (ENE). Notably, pathologists in our institution are tend to report tumors as well-moderately or moderately-poorly differentiated rather than moderately differentiated. Therefore, the differentiation status was dichotomized into moderate-poor and good-moderate level (there is no exactly moderately differentiated tumor in our cohort). For IHC staining, staining intensity was scored as follows: 0 (colorless), 1 (light yellow), 2 (brownish yellow), or 3 (brown). The IHC score equals the sum of the intensity score plus the percentage of cells of corresponding intensity (15).

### Public data retrieval and preprocessing

Publicly available HNSCC datasets were obtained from the TCGA-HNSCC. Data on RNA-seq were FPKM or TPM transformed. Genes with low expression were eliminated. After PCA, any outlier samples were removed.

### Functional characterization of differently expressed genes

The Kyoto Encyclopedia of Genes and Genomes (KEGG) database and Gene Ontology (GO) category databases were used for functional annotation of DEGs. Enrichment analysis of GO categories was performed by the R clusterProfiler (v3.14.3) package, and enrichment analysis of pathways was tested upon hypergeometric distribution by the R ‘phyper’ function. Those GO categories with an FDR<0.05 were considered significantly enriched. Pathways with a p value<0.05 were regarded as enriched. Only those GO categories or pathways containing ≥5 DEGs were kept for further analysis. For the RNA-seq data, the edgeR and DEseq2 R packages were used. Genes with an FDR<0.05 were considered differentially expressed.

### Enrichment analysis of specific gene sets

Single-sample gene set enrichment analysis (ssGSEA) was performed to calculate the enrichment score (ES) of each sample using the R package ‘GSVA’ (16) and identify up- or downregulated genes or pathways of interest in different subtypes within each tumor type by limma (17). The KEGG and biological process signature and hallmark gene sets were obtained from the Molecular Signatures Database (MSigDB, V7.2) (18). The immunologic signature was downloaded from the Immport database (19).

### Transcriptome deconvolution of the TIME

The abundance of infiltrating immune cell populations was estimated by several deconvolution methods, such as MCP (20), CIBERSORT (21), and TIMER XCELL (22). All these methods were integrated in R (immunedeconv).

### Cell culture and transient transfection

A total of two cell lines including human HNSCC SCC9 and SCC25 (the American Type Culture Collection) were used in current research. They were cultured in DMEM (Gibco, Carlsbad, CA, USA) containing 10% fetal bovine serum (Gibco, Carlsbad, CA, USA). The culture was maintained in a humidified incubator with 37°C, 5% CO2. Lipofectamine 3000 (Invitrogen, Carlsbad, CA, USA) was used to transfect Negative Control (NC) and CD36 siRNAs (GenePharma, Shanghai, China) into HNSCC cells according to the manufacturer’s instruction. Sequences for CD36 siRNAs were sense (5’-3’): GCAGCAACAUUCAAGUUAATT and antisense (5’-3’): UUAACUUGAAUGUUGCUGCTT.

### Transwell assay

The migration and invasive abilities of HNSCC cells were determined by Transwell assays (8.0 μm pores Transwell, Corning, USA) after transfection with siRNAs. Cells (1.0 × 10^5^ for migration and 2.0 × 10^5^ for invasion) were cultured in serum-free H-DMEM in the upper chambers. H-DMEM containing 10% FBS was added to the lower chambers. After cells were cultured for 24 h, cells that had migrated to the opposite side of the Transwell filter were fixed with 4% paraformaldehyde and stained with crystal violet staining solution (Beyotime, Shanghai, China). In Transwell-invasion assay, the top chamber was coated with Matrigel (1:10 in H-DMEM dilution, Corning, USA), other procedure was the same as Transwell-migration assay. Five fields were randomly selected under 100× microscope for photo recording.

### Quantitative real time PCR

Total RNA was isolated from frozen tissues and cell lines using Trizol reagent (Thermo Fisher Scientific, USA). The RNA was used to synthesize cDNA with RT Master Mix kit (TaKara, China). The qRT-PCR experiment was performed using a TB Green Premix Ex Taq Kit (TaKaRa, China) in the Applied Biosystems ViiA TM 7 Real-time PCR system (Life Technologies, CA). We used GAPDH as an internal control for normalization. The following primers were used:

CD36, forward: 5’-CTGTTATGGGGCTATAGGGATC-3’; reverse: 5’- ACTCCATCTGCAGTATTGTTGT-3’;
Snai2, forward: 5’-CTGTGACAAGGAATATGTGAGC-3’; reverse: 5’- CTAATGTGTCCTTGAAGCAACC-3’;
N-cadherin, forward: 5’- AGGAGTCAGTGAAGGAGTCAGCAG-3’; reverse: 5’- TTCTGGCAAGTTGATTGGAGGGATG-3’;
Vimentin, forward: 5’- CCTTCGTGAATACCAAGACCTGCTC-3’; reverse: 5’- AATCCTGCTCTCCTCGCCTTCC-3’;
S100A8, forward: 5’-CTAATGTGTCCTTGAAGCAACC-3’; reverse: 5’- TCTGCACCCTTTTTCCTGATAT-3’
S100A9, forward: 5’- CCTTCCACCAATACTCTGTGAA-3’; reverse: 5’- GGTCCTCCATGATGTGTTCTAT-3’

### Statistical analysis

The ^18^F-FDG PET/CT interpretations were compared with the final pathology reports in analyses based on patient as we illustrated previously.

For categorical variables, we made comparison between pathologically positive and negative groups (**Table 1**), FN and TN groups (**Table 2**), and FN and TP groups (**Table 3**), which were analyzed by the Chi-squared test or Fisher’s exact test. For continuous variables with a skewed distribution (**Table S1**; **Table S2**), a nonparametric Mann–Whitney U test was used. The Spearman correlation coefficient was used to assess the correlation between two IHC scores. A two-tailed p-value less than 0.05 was considered that the difference was statistically significant. SPSS software (SPSS version 24.0; SPSS Inc., Chicago, IL, United States) was used to perform statistical analysis.

**Table 1:** Overview of clinical factors of patients and association of clinical factors with lymph node metastasis.

Characteristic	n	False negative	True positive	True negative	False positive	Pathologically positive	Pathologically negative	*P* value
Sex								
Female	28(30.43%)	1	12	9	6	13	15	0.250
Male	64(69.57%)	12	26	15	11	38	26	
Age								
<60	31(33.70%)	4	15	8	4	19	12	0.421
≥60	61(66.30%)	9	23	16	13	32	29	
pT								
T1-T2	44(47.83%)	0	18	16	10	18	26	0.007^**^
T3-T4	48(52.17%)	13	20	8	7	33	15	
pN								
N0-N1	52(56.52%)	4	7	24	17	11	41	<0.001^***^
N2-N3	40(43.48%)	9	31	0	0	40	0	
Clinical stage								
I-II	26(28.26%)	0	0	16	10	0	26	<0.001^***^
III-IV	58(73.42%)	13	38	8	7	51	15	
Neuron/vessel invasion								
Positive	24(26.09%)	7	10	5	2	17	7	0.078
Negative	68(73.91%)	6	28	19	15	34	34	
DOI								
≤10mm	45(48.91%)	1	19	15	10	20	25	0.038^*^
>10mm	47(51.09%)	12	19	9	7	31	16	
Differentiation status								
Moderate-poor/poor	62(67.39%)	11	23	17	11	34	28	0.869
Good/good-moderate	30(32.61%)	2	15	7	6	17	13	
Primary lesion site								
Tongue	48(52.17%)	9	21	12	6	30	18	NA
Gingiva	14(15.22%)	1	4	4	5	5	9	
Buccal	6(6.52%)	0	2	3	1	2	4	
Palate	6 (6.52%)	0	5	0	1	5	1	
Floor of mouth	10(10.87%)	2	3	4	1	5	5	
Others	8 (8.7%)	1	3	1	3	4	4	
SUV_max_ of primary lesion								
<12.76	47(51.00%)	6	14	18	9	20	27	0.019^*^
≥12.76	45(48.91%)	7	24	6	8	31	14	
Extranodal extension (ENE)								
positive	20(39.22%)	5	15	NA	NA	20	NA	NA
negative	31(60.78%)	8	23	NA	NA	31	NA	

Pathologically positive group vs pathologically negative group, p values were calculated withχ^2^ or Fisher’s exact test.

* p value<0.05, ** p value <0.01, *** p value <0.001. NA, not applicable.

**Table 2:** Clinical characteristics of false negative group compared to true negative group.

Characteristic	n	False negative	True negative	*P* value
Primary lesion site				
Tongue	21 (56.76%)	9	12	0.315
Others	16 (43.24%)	4	12	
SUV_max_ of primary lesion				
<12.76	24 (64.86%)	6	18	0.079
≥12.76	13 (35.14%)	7	6	
DOI				
≤10mm	16 (43.24%)	1	15	0.002^**^
>10mm	21 (56.76%)	12	9	
pT				
T1-T2	16 (43.24%)	0	16	<0.001^***^
T3-T4	21 (56.76%)	13	8	
Neuron/vessel invasion				
Positive	12 (32.43%)	7	5	0.067
Negative	25 (67.57%)	6	19	
Differentiation status				
Moderate-poor/poor	28 (75.68%)	11	17	0.446
Good/good-moderate	9 (24.32%)	2	7	

False negative group vs true negative group, p values were calculated withχ2 or Fisher’s exact test. ** p value <0.01; *** p value <0.001

**Table 3:** Clinical characteristics of false negative group compared to true positive group.

Characteristic	n	False negative	True positive	*P* value
Primary lesion site				
Tongue	30 (58.82%)	9	21	0.518
Others	21 (39.22%)	4	17	
SUV_max_ of primary lesion				
<12.76	20 (39.22%)	6	14	0.553
≥12.76	31 (60.78%)	7	24	
DOI				
≤10mm	20 (39.22%)	1	19	0.008^**^
>10mm	31 (60.78%)	12	19	
pT				
T1-T2	18 (35.29%)	0	18	0.002^**^
T3-T4	33 (64.71%)	13	20	
Neuron/vessel invasion				
Positive	17 (33.33%)	7	10	0.069
Negative	34 (66.67%)	6	28	
Differentiation status				
Moderate-poor/poor	34 (66.67%)	11	23	0.175
Good/good-moderate	17 (33.33%)	2	15	

False negative group vs true positive group, p values were calculated withχ^2^ or Fisher’s exact test.

** p value <0.01.

## Results

### Description of the study population

The patient cohort consisted of 92 patients (female n=28, male n=64) with primary diagnosed HNSCC whose average age was 62 ± 12 years. **Table 1** presents the clinical characteristics of patients. The most common primary tumor site was the tongue (52.17%), followed by the gingiva (15.22%), floor of mouth (10.87%), buccal (6.52%), and palate (6.52%). The tumor was well (well-moderately) and poorly (moderately-poorly) differentiated in 30 (32.61%) and 62 (67.39%) patients. 48 patients (52.17%) had stage pT3–pT4 disease, 24 (26.09%) had positive neuron/vessel invasion findings, and 20 (39.22% in patients with lymph node metastasis) exhibited ENE.

Then, we investigated the association of these clinical factors and lymph node metastasis. We found a significant association of advanced pT stage (T3-T4 vs. T1-T2, *p*=0.007), advanced clinical stage (III-IV stage vs. I-II stage, *p*<0.001), and larger depth of invasion (DOI) (>10 mm vs. ≤10 mm, *p*=0.038) with lymph node metastasis, which was consistent with current studies since these indexes represented invasion and metastasis capability of tumor cells.

Since primary tumor ^18^F-FDG uptake was rarely considered in lymph node metastasis evaluation, we wondered whether they had somewhat association. Unluckily, there was no criteria of primary tumor SUV_max_ for lymph node metastasis diagnosis yet, so we used median SUV_max_ (median SUV_max_ =12.76 of our cohort) as substitutive cut-off value. We compare lymph node metastasis between high-SUV_max_ and low-SUV_max_ group and found that larger SUV_max_ of primary lesions (≥ 12.76 vs. <12.76, *p*=0.019) were associated with lymph node metastasis, which suggested that metabolic activity of tumor cells in primary tumor may refer to their malignant capability to some extent. This result confirmed the viability of our study which was aim to distinguish FN lymph nodes with metabolic information of primary tumors.

### Association between clinical factors and false-negative ^18^FDG-PET/CT results of lymph nodes

To identify which clinical factors may be associated with ^18^FDG-PET/CT FN diagnosis, we reviewed the clinical characteristics of the FN, TN, and TP groups.

When comparing the FN group with the TN group (**Table 2**), we found that FN were associated with a larger DOI (>10 mm vs. ≤10 mm, *p*=0.002) and advanced pT stage (T3-T4 vs. T1-T2, *p*<0.001) of the primary lesion. Interestingly, when we compared the FN group with the TP group (**Table 3**), we also found that FN lymph nodes were associated with a larger DOI (>10 mm vs. ≤10 mm, *p*=0.008) and advanced pT stage (T3-T4 vs. T1-T2, *p*=0.002) of the primary lesion, which indicated that those primary tumors with metastatic lymph nodes not being detected by ^18^FDG-PET/CT may exhibit higher degree of malignancy.

Moreover, when comparing FN with TN (≥ 12.76 vs. <12.76, *p*=0.079) or FN with TP (≥ 12.76 vs. <12.76, *p*=0.553), diagnosis had no association with the SUV_max_ of the primary lesion, which suggested that though ^18^F-FDG uptake of the primary lesion was associated with lymph node metastasis (**Table 1**), this index was unable to distinguish FN from TN or TP. Based on these results, we were interested in the exact association between metabolic patterns of primary tumor with lymph node status and try to find a more solid metabolic marker to distinguish FN lymph nodes.

### CD36 and GLS expression in primary lesions is related to false negative ^18^FDG-PET/CT results in lymph nodes

**Figure 2:**
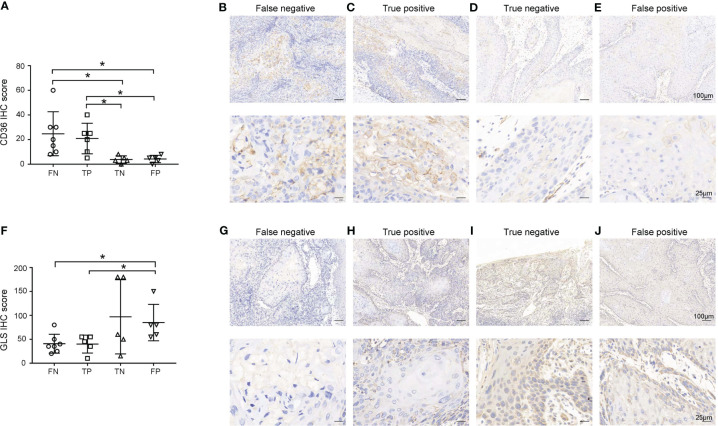
Expression of CD36 **(A-E)** and GLS **(F-J)** in primary lesions. **(A, F)** Expression of CD36 **(A)** and GLS **(F)** in primary lesions in the false negative (FN), true positive (TP), true negative (TN), and false positive (FP) groups. **(B-E, G-J)** Representative images of each group are shown. Scale bar in the upper line: 100μm, lower line: 25μm. *p < 0.05.

**Figure 3:**
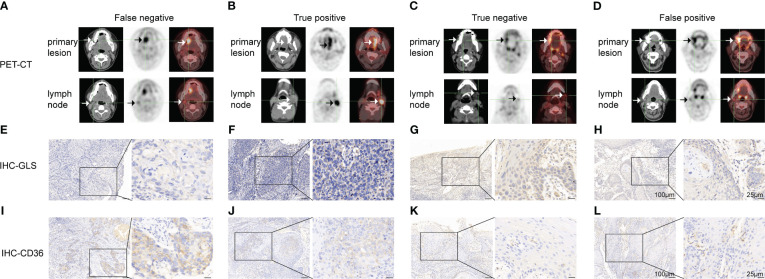
Representative cases of false negative **(A, E, I),** true positive **(B, F, J)**, true negative **(C, G, K)** and false-positive **(D, H, L)** groups. **(A-D)** ^18^FDG-PET/CT pictures of the primary lesion and lymph node. **(E-H)** GLS IHC images of the primary lesion. **(I-L)** CD36 IHC images of the primary lesion. Scale bar: left panel: 100μm, right panel: 25μm.

The ^18^FDG-PET/CT examination makes use of the abnormal glucose uptake of tumor cells; therefore, we hypothesized that tumors of FN group exhibited different metabolic patterns from other groups. Thus, we examined the expression of GLUT1, GLUT5, GLS, SLC1A5, CPT1A, and CD36, which were key molecules involved in three major metabolism, of primary lesions in four groups. As shown in **Figure 2**; **Table S2**, the CD36 IHC score of primary lesions was significantly higher in the pathologically positive group than the pathologically negative group (FN vs. TN *p*=0.028, FN vs. FP *p*=0.031, TP vs. TN *p*=0.016, TP vs. FP *p*=0.018) while the GLS IHC score of primary lesions was significantly lower in FN and TP group than FP group (FN vs. FP *p*=0.025, TP vs. FP *p*=0.045). However, GLUT1 of primary tumors seemed to lose predictive value since large difference within groups (data not shown). Representative case pictures are shown in **Figure 3**. These results indicated that by preoperative biopsy of the primary lesion and IHC examination of CD36 and GLS, we are likely to exclude ^18^FDG-PET/CT FN diagnosis of lymph nodes.

The association of the IHC scores of GLUT1, GLUT5, GLS, SLC1A5, CPT1A, and CD36 in primary lesions with clinical factors is shown in **Table S1**; **Figure S2**.

### Metabolic rewiring of metastasis lymph nodes

**Figure 4:**
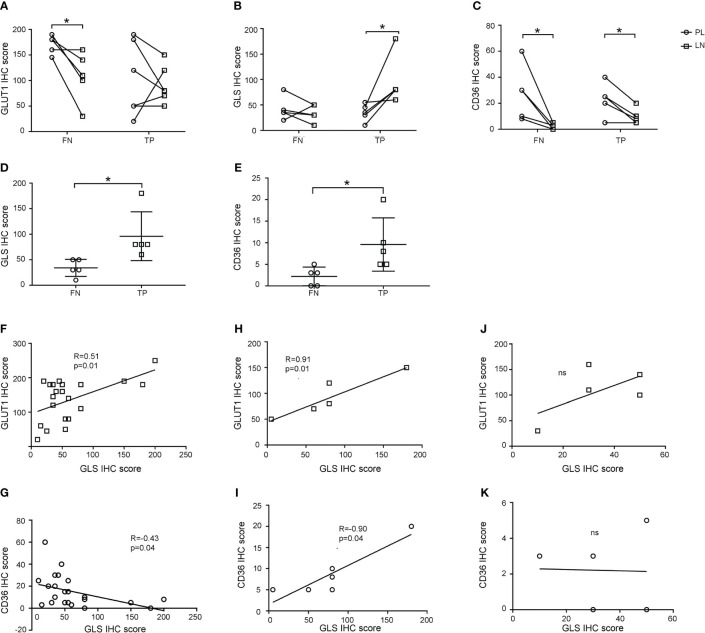
Expression patterns of GLUT1, GLS, and CD36 in primary lesions and lymph nodes. **(A-C)** GLUT1 **(A)**, GLS **(B)**, and CD36 **(C)** expression in primary lesions and lymph nodes in false negative and true positive groups. **(D, E)** Comparison of GLS **(D)** and CD36 **(E)** IHC scores between false negative and true positive nodes. **(F-K)** Correlation of GLS and GLUT1 **(F, H, J)** and GLS and CD36 **(G, I, K)** IHC scores in primary lesions **(F, G)**, true positive nodes **(H-I)** and false negative nodes **(J, K)**. *p < 0.05. ns, not significant.

Next, we investigated the expression of GLUT1, GLUT5, GLS, SLC1A5, CPT1A, and CD36 in lymph nodes and compared them with those in primary lesions (representative pictures are shown in **Figure S3**). As shown in **Figures 4A-C**, the GLUT1 IHC score in the lymph nodes was equal to that in the primary lesion in TP group, while the GLS IHC score (*p*=0.046) increased and the CD36 IHC score (*p*=0.022) decreased in the lymph nodes. Meanwhile, for the FN group, GLUT1 (*p*=0.034) and CD36 (*p*=0.044) IHC scores both decreased in lymph nodes, while GLS IHC scores showed no significant change. These results indicated that tumor cells underwent a metabolic rewiring for metastasizing to lymph nodes and that the metabolic transition pattern of the FN group was different from that of the TP group: the overall metabolic activity of the FN group was weakened in the lymph nodes. Moreover, we found that GLS and CD36 IHC scores in FN nodes were significantly lower than those in TP nodes (**Figures 4D, E**, *p*=0.025 and 0.036, respectively).

We investigated the metabolic rewiring pattern further by calculating the correlation of the IHC scores of GLUT1, GLUT5, GLS, SLC1A5, CPT1A, and CD36. In primary lesions, the GLUT1 IHC score was positively correlated with GLS (R=0.51, *p*=0.01), and CD36 was negatively correlated with GLS (R=-0.43, *p*=0.04) (**Figures 4F, G**), which represented the canonical characteristics of tumor cell metabolism in primary lesions. When looking into lymph nodes, metabolic markers still kept correlation in TP group as shown in **Figures 4H, I** (GLS with GLUT1: R=0.91, *p*=0.01, GLS with CD36: R=0.90, *p*=0.01), but lost their correlation in FN group as shown in **Figures 4J, K**, which suggested that tumor cells in lymph nodes in FN groups may have noncanonical metabolic pattern.

In conclusion, these results suggested that tumor cells underwent metabolic reprogramming in metastatic lymph node. Malignant cells in FN nodes had lower metabolic activity than those in TP nodes and that amino acid and lipid metabolism were promising targets for developing new clinical examinations of lymph node metastasis.

### CD36 is related to tumor invasive characteristic

**Figure 5:**
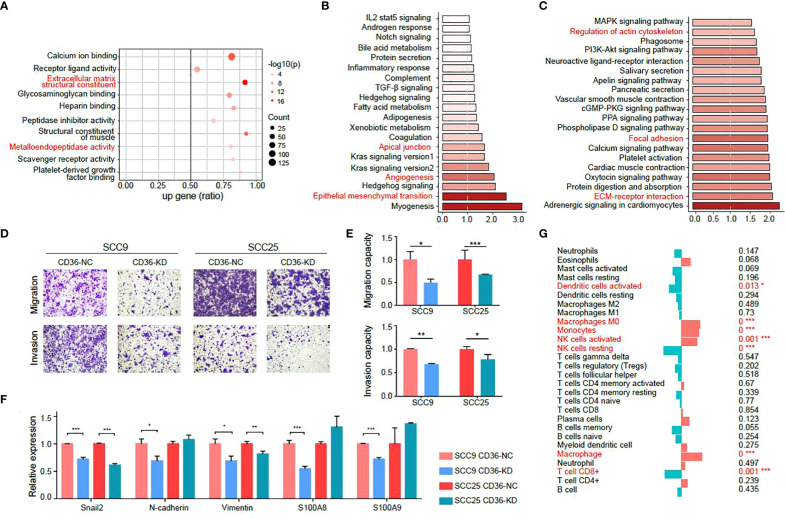
CD36 is associated with aggressive characteristics of tumor cells. **(A-C)** GO **(A)**, GSEA **(B)** and KEGG **(C)** analysis of CD36 in the TCGA-HNSCC database. **(D-E)** Images of Transwell assays for migration and invasion **(D)** in negative control and CD36 knockdown group. The quantitative analysis of migration and invasion ability is shown in **(E)**. **(F)** Differences in expression of EMT-related genes between negative control and CD36 knockdown group. **(G)** Association of CD36 expression and immune infiltration. *p < 0.05, **p < 0.01, ***p < 0.001.

As CD36 was demonstrated to promote tumor metastasis, we will focus on CD36 in the following study. First, we turned to a public database to determine biological activities related to CD36. By analyzing the TCGA-HNSCC database (**Figures 5A-C**), we found that CD36 was related to “extracellular matrix structural constituents”, “metalloendopeptidase activity”, “apical junctions”, and “epithelial mesenchymal transition”, all of which enriched in metastasis-promoting functions.

Then, we validated the metastasis promoting characteristics of CD36 *in vitro* in two oral cancer cell lines: SCC9 and SCC25, both of which exhibit relatively high endogenous CD36 levels (**Figure S4A**). We knocked down CD36 in SCC9 and SCC25 cells (**Figure S4B**) and observed CD36 silencing significantly inhibited aggressive behaviors of oral cancer cells by transwell migration and invasion assay (**Figures 5D, E**). The PCR results showed that expression levels of EMT markers and genes related to metastasis such as S100A8 and S100A9 were significantly decreased after knocking down CD36 in oral cancer cells (**Figure 5F**).

Moreover, when analyzing immune infiltration status, we found that the CD36 expression level was negatively related to CD8^+^ T cells, which indicated that CD36^+^ tumor cells could promote tumor progression by exhibiting immune infiltration (**Figure 5G**).

Collectively, we concluded metastasis-promoting characteristics of CD36 by investigating public databases and validated that by experiments *in vitro*.

## Discussion

Lymph node metastasis frequently occurs in HNSCC, influences the clinical therapy plan and poses an unfavorable factor for patient outcomes. Although ^18^FDG-PET/CT is considered a good examination method to identify lymph node metastasis, the false negative problem is also unavoidable. Previous studies on this topic mainly focused on comparing different diagnostic methods (3, 5) or evaluating the influences of diagnosis results on clinical decisions (4, 6). However, few, if any, researchers analyzed primary lesions for a biomarker of false negative patients.

In our study, we performed a histopathological examination of GLUT1, GLUT5, GLS, SLC1A5, CPT1A, and CD36 expression in primary lesions as well as lymph nodes from HNSCC patients. CD36 in the primary lesion showed higher expression, while GLS showed lower expression in FN group than TP group.

GLS is the enzyme critical for glutamine utilization. Glutamine addiction is common in cancer cells, and GLS was reported to promote cancer cell proliferation and invasion (23, 24). However, the correlation of GLS with lymph node metastasis is still unclear, and the results in existing articles are inconsistent (23, 25). In our study, we found that in patients with lymph node metastasis, GLS expression in their primary lesion was significantly lower than that in patients with no metastasis. The mechanism behind this phenomenon requires further exploration.

As for CD36, a scavenger receptor expressed in multiple cell types mediating lipid uptake, a series of studies have demonstrated its metastasis-promoting functions (13, 26–29). For example, in ovarian cancer, CD36 facilitates adaption and metastasis of tumor cells in adipocyte-rich TME (30). In gastric cancer, palmitate acid (PA) induces cancer cells to upregulate CD36, activating downstream pro-tumor signaling pathways (31, 32). PA was also found to promote CD36-mediated metastasis in HNSCC (33). Moreover, recent studies also unraveled pro-tumor roles of CD36 on immune cells in the TME. Yang. Et.al observed that metastasis-associated macrophages (MAM) upregulate CD36, which fuel macrophage-tumor crosstalk and reshape the TME (34). In TME of hepatocellular carcinoma (HCC), a subcluster of cancer-associated fibroblast (CAF) expressed CD36 exhibited high-level lipid metabolism and expression of macrophage migration inhibitory factor (MIF), which interacted with HCC and myeloid-derived suppressor cells to provide immunosuppressive microenvironment (35). However, despite of quantities of molecular biology studies on CD36, it has not been applied with ^18^FDG-PET/CT for distinguishing false negatives yet. We validated that CD36 expression in primary lesions was higher in FN patients than TN patients. This meant that by preoperative biopsy and histopathological examination of CD36, we could distinguish patients with lymph node metastasis, although their ^18^FDG-PET/CT showed N0 results.

Since we only had retrospective paraffin samples of the ^18^FDG-PET/CT cohort, we tried to further explore metabolic characteristics of primary lesions and lymph nodes between groups by analyzing metabolic marker expression based on IHC. We found that GLUT1, GLS, and CD36 expression levels were significantly changed between them, and FN nodes showed an overall lower metabolic activity than TP nodes. This may be one of the causes for FN results in ^18^FDG-PET/CT examinations. These results inspired us to use IHC for confirming lymph node metastasis status beyond ^18^FDG-PET/CT examinations.

Compared to GLS, there are more studies focused on CD36 and tumor metastasis, so we validated this marker in oral cancer cell lines *in vitro*. Transwell migration and invasion assays showed that when CD36 was knockdown in cancer cells, their migration and invasion abilities were significantly impaired. Epithelial-mesenchymal transition is a hallmark of cancer metastasis, whose marker genes includes snai2, N-cadherin and vimentin (36). The expression of these three genes decreased after CD36 knockdown. Also, S100A8 and S100A9 were down-regulated after CD36 knockdown, which are classical ligands related to cancer aggressiveness (37, 38).

The following shortcomings of the present study need to be discussed.

First, the significance of our study lies in clinical translation so we did not pay close attention to the molecular mechanism. Second, since the nuclear medicine department in our hospital was not established for a long time and the cases included in our study were limited. We can only acquire retrospective paraffin samples, which means that novel sequencing methods and multiomics technology are not allowed to use. We will design a prospective study to consolidate the significance of CD36 and GLS in distinguishing false negatives in subsequent studies with the aid of high-throughput sequencing and multiomics technologies. Moreover, we did not include other imaging methods into consideration. For example, magnetic resonance imaging (MRI) has been used for assessing lymph node metastasis in HNSCC patients and showed satisfying results (39). It would be meaningful to design a clinical study comparing efficiency in diagnosing lymph node metastasis between MRI and ^18^FDG-PET/CT.

## Conclusion

CD36 expression was higher in the ^18^FDG-PET/CT false-negative group than in the pathologically negative group. The correlation of CD36 expression with invasive biological characteristics of HNSCC was validated in both bioinformatics analysis and *in vitro* experiments. Therefore, CD36 could be promising biomarker for primary lesion biopsy to detect false-negative lymph nodes in ^18^FDG-PET/CT.

## Data availability statement

The original contributions presented in the study are included in the article/**Supplementary Material**. Further inquiries can be directed to the corresponding authors.

## Ethics statement

The studies involving human participants were reviewed and approved by Ethics Committee of Shanghai Ninth People’s Hospital Affiliated to Shanghai Jiao Tong University School of Medicine. The patients/participants provided their written informed consent to participate in this study. Written informed consent was obtained from the individual(s) for the publication of any potentially identifiable images or data included in this article.

## Author contributions

YH and ZL conceived and carried out experiments. XM, JS, and FX conceived experiments and analyzed data. XM carried out experiments. All authors were involved in writing the paper and had final approval of the submitted and published versions. All authors contributed to the article and approved the submitted version.
