# Supplementary material for: Lipid metabolism marker CD36 is associated with 18FDG-PET/CT false negative lymph nodes in head and neck squamous cell carcinoma
Source: Front Oncol. 2023 May 3;13:1156527. doi: 10.3389/fonc.2023.1156527 (PMC10189774; doi:10.3389/fonc.2023.1156527)
Supplement: Supplementary file 1 [file DataSheet_1.pdf]

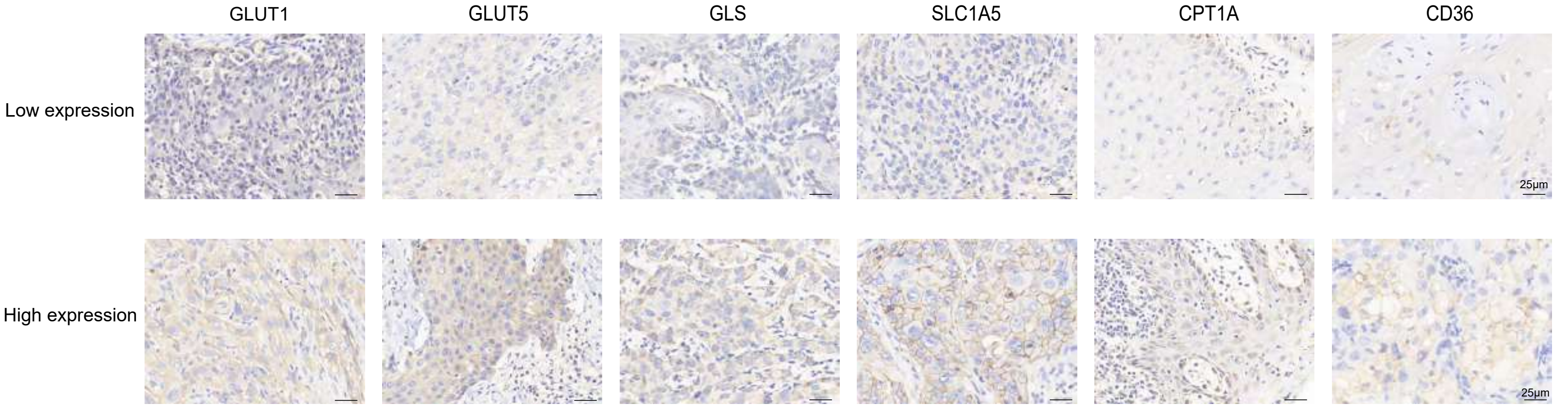
GLUT1
GLUT5
GLS
SLC1A5
CPT1A
CD36
Low expression
High expression
25μm
25μm

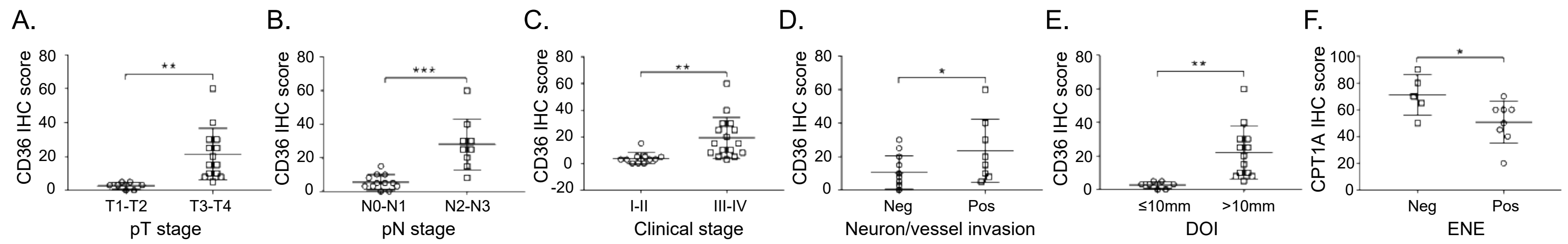

A.
CD36 IHC score
80
60
40
20
0
**
T1-T2
T3-T4
pT stage
B.
CD36 IHC score
80
60
40
20
0
***
N0-N1
N2-N3
pN stage
C.
CD36 IHC score
80
60
40
20
0
-20
**
I-II
III-IV
Clinical stage
D.
CD36 IHC score
80
60
40
20
0
*
Neg
Pos
Neuron/vessel invasion
E.
CD36 IHC score
80
60
40
20
0
**
≤10mm
>10mm
DOI
F.
CPT1A IHC score
100
80
60
40
20
0
*
Neg
Pos
ENE

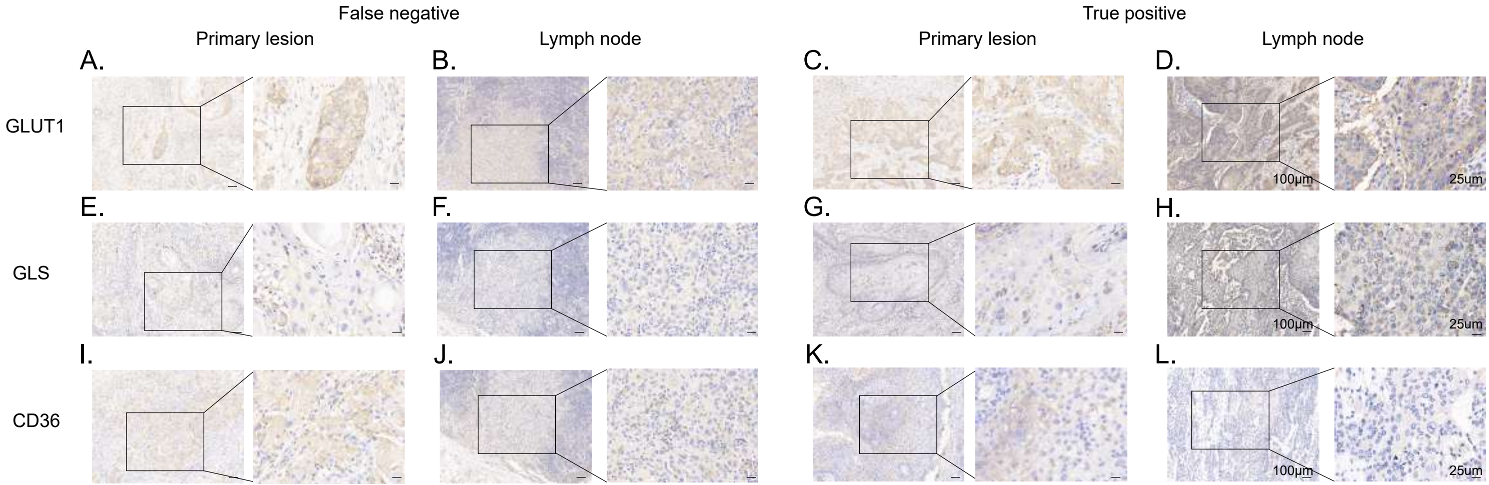
False negative
True positive
Primary lesion
Lymph node
Primary lesion
Lymph node
A.
B.
C.
D.
GLUT1
E.
F.
G.
H.
GLS
I.
J.
K.
L.
CD36
100μm
25um
100μm
25um
100μm
25um

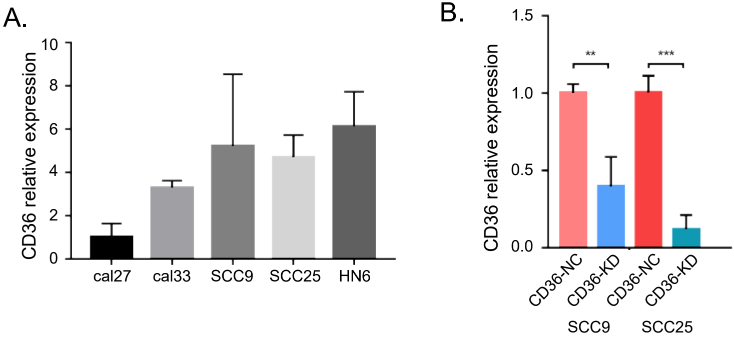

A.
CD36 relative expression
10
8
6
4
2
0
cal27
cal33
SCC9
SCC25
HN6
B.
CD36 relative expression
1.5
1.0
0.5
0.0
**
***
CD36-NC
CD36-KD
CD36-NC
CD36-KD
SCC9
SCC25

Figure S1. Tissue sections stained for GLUT1, GLUT5, GLS, SLC1A5, CPT1A, and CD36. GLUT1, GLUT5, SLC1A5, and CD36 were stained mainly in the membrane, while GLS and CPT1A showed Cytoplasmic positivity. Representation of low expression (upper line) and high expression (below line). Scale bar: 25μm.

Figure S2. Association of metabolic markers with clinical factors. (A) The CD36 IHC score was higher in advanced pT stage (T3-T4 vs. T1-T2, $p=0.003$). (B) The CD36 IHC score was higher in advanced pN stage (N2-N3 vs. N0-N1, $p<0.001$). (C) The CD36 IHC score was higher in advanced clinical stage (III-IV stage vs. I-II stage, $p=0.005$). (D) The CD36 IHC score was higher in the neuron/vessel invasion-positive group (positive vs. negative, $p=0.040$). (E) The CD36 IHC score was higher in the larger DOI group (>10 mm vs. ≤ 10 mm, $p=0.006$). (F) The CPT1A IHC score was higher in the ENE-negative group (negative vs. positive, $p=0.026$).
.
Figure S3. Expression of GLUT1 (A-D), GLS (E-H), and CD36 (I-L) in primary lesions (A, C, E, G, J, K) and lymph nodes (B, D, F, H, J, L). Scale bar: left panel: 100μm, right panel: 25μm.

Figure S4. (A) Expression of CD36 in different oral cancer cell lines. CD36 expression level in cal27 was used as control. (B) Quantitative real time PCR results validated that siRNA successfully knockdown CD36 expression in SCC9 and SCC25 cell lines.
