# Supplementary material for: Lipid metabolism marker CD36 is associated with 18FDG-PET/CT false negative lymph nodes in head and neck squamous cell carcinoma
Source: Front Oncol. 2023 May 3;13:1156527. doi: 10.3389/fonc.2023.1156527 (PMC10189774; doi:10.3389/fonc.2023.1156527)
Supplement: Supplementary file 2 [file Table_1.docx]

**Supplementary table1** Association between clinical factors with IHC scores of metabolic markers of primary tumors

|  | **GLUT1** | **GLUT5** | **GLS** | **SLC1A5** | **CPT1A** | **CD36** |
| --- | --- | --- | --- | --- | --- | --- |
| **Age** | | | | | | |
| **<60** | 117.27±63.89 | 53.64±50.60 | 52.27±40.09 | 75.91±47.37 | 53.27±24.92 | 53.25±27.42 |
| **≥60** | 150.83±57.40 | 60.00±48.19 | 72.50±56.99 | 86.67±60.05 | 48.50±28.96 | 45.45±11.44 |
| **pT** | | | | | | |
| **T1-T2** | 115.56±51.02 | 60.56±57.25 | 65.00±46.97 | 61.11±49.86 | 43.00±33.89 | 4.33±4.44 |
| **T3-T4** | 147.14±66.35 | 54.64±43.83 | 61.43±52.93 | 94.64±53.15 | 55.79±20.54 | 21.86±15.04^***^ |
| **pN** | | | | | | |
| **N0-N1** | 138.46±60.08 | 58.08±46.93 | 80.00±59.02 | 77.69±57.90 | 48.31±33.16 | 6.69±5.84 |
| **N2-N3** | 130.00±66.50 | 55.50±52.62 | 40.50±19.78 | 86.50±49.56 | 54.00±15.60 | 25.80±16.01^***^ |
| **Clinical stage** | | | | | | |
| **I-II** | 115.56±51.02 | 60.56±57.25 | 65.00±46.97 | 61.11±49.86 | 43.00±33.89 | 4.33±4.44 |
| **III-IV** | 147.14±66.35 | 54.64±43.83 | 61.43±52.93 | 94.64±53.15 | 55.79±20.54 | 21.86±15.04^***^ |
| **Neuron/vessel invasion** | | | | | | |
| **Positive** | 166.88±27.64 | 75.00±48.99 | 56.88±41.40 | 107.50±56.51 | 61.38±12.91 | 23.50±18.88 |
| **Negative** | 117.67±68.42 | 47.33±46.75 | 66.00±54.58 | 67.67±47.92 | 45.13±30.50 | 10.47±9.99^*^ |
| **DOI** | | | | | | |
| **≤10mm** | 108.50±53.02 | 58.00±54.58 | 61.00±46.06 | 60.00±47.14 | 47.70±35.24 | 5.90±6.49 |
| **>10mm** | 155.00±61.91 | 56.15±45.24 | 64.23±54.00 | 98.08±53.68 | 53.15±18.76 | 22.00±15.64^**^ |
| **Differentiation status** | | | | | | |
| **Moderate-poor/poor** | 92.50±44.25 | 75.00±79.37 | 41.25±20.56 | 52.50±22.17 | 51.75±40.48 | 10.25±8.62 |
| **Good/good-moderate** | 143.68±61.91 | 53.16±41.44 | 67.37±53.03 | 87.63±56.38 | 50.58±24.32 | 16.00±15.72 |
| **SUV_max_ of primary lesion** | | | | | | |
| **<12.76** | 150.45±57.55 | 53.64±26.56 | 57.73±50.56 | 74.55±45.91 | 51.82±26.86 | 18.36±18.94 |
| **≥12.76** | 120.42±64.12 | 60.00±63.32 | 67.50±50.48 | 87.92±60.81 | 49.83±27.52 | 11.92±9.32 |
| **Extranodal extension (ENE)** | | | | | | |
| **Positive** | 132.50±65.19 | 57.50±58.25 | 38.13±21.54 | 81.25±54.36 | 50.63±15.68 | 24.13±17.25 |
| **Negative** | 136.00±61.94 | 56.67±44.43 | 76.00±55.68 | 81.67±54.83 | 50.87±31.45 | 10.13±10.94^*^ |

*p* values were calculated with nonparametric Mann–Whitney U test.

**p* value<0.5, ** *p* value<0.1, *** *p* value<0.001

**Supplementary table2** IHC scores of metabolic markers of primary tumor in different groups

|  | **TP** | **FN** | **FP** | **TN** |
| --- | --- | --- | --- | --- |
| **GLUT1** | 101.67±72.50 | 151.43±50.72 | 128.00±53.57 | 158.00±68.70 |
| **GLUT5** | 57.50±68.68 | 73.57±51.86 | 36.00±35.95 | 54.00±24.08 |
| **GLS** | 38.33±17.22 | 40.71±19.88 | 85.00±38.08 | 101.00±83.25 |
| **SLC1A5** | 102.50±49.77 | 87.14±57.94 | 56.00±63.87 | 74.00±43.36 |
| **CPT1A** | 58.33±12.91 | 58.57±22.68 | 28.20±27.50 | 53.40±36.30 |
| **CD36** | 21.67±11.69 | 24.71±17.86 | 4.60±3.65 | 3.80±2.95 |

TP: true positive; FN: false negative; FP: false positive; TN: true negative
